# Mitochondrial fusion–fission dynamics and its involvement in colorectal cancer

**DOI:** 10.1002/1878-0261.13578

**Published:** 2024-01-09

**Authors:** Zihong Wu, Chong Xiao, Fang Li, Wenbo Huang, Fengming You, Xueke Li

**Affiliations:** ^1^ Hospital of Chengdu University of Traditional Chinese Medicine China; ^2^ Oncology Teaching and Research Department Chengdu University of Traditional Chinese Medicine China; ^3^ Institute of Oncology Chengdu University of Traditional Chinese Medicine China

**Keywords:** colorectal cancer, dynamics, mitochondrial fission, mitochondrial fusion, progression

## Abstract

The incidence and mortality rates of colorectal cancer have elevated its status as a significant public health concern. Recent research has elucidated the crucial role of mitochondrial fusion–fission dynamics in the initiation and progression of colorectal cancer. Elevated mitochondrial fission or fusion activity can contribute to the metabolic reprogramming of tumor cells, thereby activating oncogenic pathways that drive cell proliferation, invasion, migration, and drug resistance. Nevertheless, excessive mitochondrial fission can induce apoptosis, whereas moderate mitochondrial fusion can protect cells from oxidative stress. This imbalance in mitochondrial dynamics can exert dual roles as both promoters and inhibitors of colorectal cancer progression. This review provides an in‐depth analysis of the fusion–fission dynamics and the underlying pathological mechanisms in colorectal cancer cells. Additionally, it offers partial insights into the mitochondrial kinetics in colorectal cancer‐associated cells, such as immune and endothelial cells. This review is aimed at identifying key molecular events involved in colorectal cancer progression and highlighting the potential of mitochondrial dynamic proteins as emerging targets for pharmacological intervention.

## Graphical abstract

Disruption of the mitochondrial fusion–fission dynamics balance plays a dual role in colorectal cancer development. Mitochondrial fission promotes cancer cell proliferation, invasion, migration, and chemoresistance, while excessive fission induces apoptosis. Similarly, mitochondrial fusion attenuates intestinal epithelial cells from oxidative stress damage, whereas excessive fusion promotes abnormal tumor proliferation and metastasis.

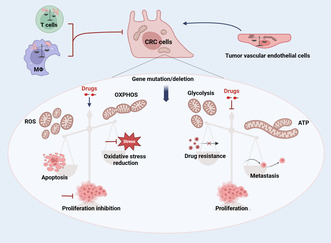

**Abbreviations**
AGP: *Aloe gel* glucomannan
AIK: apoptosis inducer kit
CAC: colitis‐associated cancer
CCSCs: colorectal cancer stem cells
CHD6: chromodomain helicase DNA binding protein 6
CRC: colorectal cancer
DRP1: dynamin‐related protein 1
ER: endoplasmic reticulum
ETC: electron transport chain
FAs: fatty acids
HB: helix bundle
IMM: inner mitochondrial membrane
LLPs: liquid–liquid phase separation
MCU: mitochondrial calcium uniporter
MFF: mitochondrial fission factor
MFN: mitofusin
MMP: mitochondrial membrane potential
mPTP: mitochondrial permeability transition pore
MTFR2: mitochondrial fission regulator 2
OMM: outer mitochondrial membrane
OPA1: optic atrophy 1
OPWPs: oil production waste products
OXPHOS: oxidative phosphorylation
PDK1: pyruvate dehydrogenase kinase 1
ROS: reactive oxygen species
TCA: tricarboxylic acid cycle

## 1 Introduction

Colorectal cancer (CRC) is a major concern as it ranks as the third most commonly diagnosed cancer in both males and females and is the second leading cause of cancer‐related deaths in the United States [1]. The incidence of CRC is increasing among young adults, making it an increasingly pressing social issue [2]. Despite the advances in treatment technology, CRC remains one of the deadliest cancer types due to the challenges associated with metastasis and drug resistance. Therefore, developing a comprehensive understanding of the critical molecular events in CRC progression and identifying new targets for therapy are crucial. Current tumor research primarily focuses on exploring the relationship between metabolic changes and human cancers. In the case of CRC, it is specifically characterized by mitochondrial dysfunction resulting in decreased respiration but increased glycolysis [3].

Mitochondria are widely recognized as the “energy factories” of eukaryotic cells due to their ability to generate ATP. Moreover, they play pivotal roles in cell differentiation, apoptosis, calcium homeostasis, innate immunity, and the metabolism of fatty acids (FAs) and amino acids [4, 5]. These dynamic organelles are transported along the cytoskeleton and exhibit various morphologies. Their collective activity is regulated through processes of fusion and fission, enabling the merging of shorter tubules into longer structures and the division of tubules into smaller spheres [6]. The continuous interplay of mitochondrial membrane fusion and fission contributes to the regulation of mitochondrial morphology and quantity, ensuring their homogeneity and efficient function [6, 7]. Additionally, mitophagy, another aspect of mitochondrial dynamics, is a crucial mechanism in mitochondrial quality control as it selectively eliminates dysfunctional mitochondria [8].

The term “mitochondrial dynamics” encompasses various processes, including fission, fusion, mitophagy, transport, and interactions with other organelles such as the endoplasmic reticulum (ER). While mitophagy is a component of mitochondrial dynamics and plays a key role in CRC biology, it has been extensively discussed elsewhere [5, 9]. Therefore, this review focuses on the role of mitochondrial fusion–fission dynamics in CRC cells. We begin with a brief overview of the machinery involved in mitochondrial fusion and fission, followed by a comprehensive review of the relationship between altered fusion–fission dynamics in CRC cells and tumor development. Additionally, we mention the imbalance of mitochondrial dynamics in immune and vascular endothelial cells, underscoring its potential clinical value in the treatment of CRC.

## 2 Mitochondrial dynamics: fusion and fission

### 2.1 Mechanisms of mitochondrial fusion

Mitochondria consist of two membranes: the outer mitochondrial membrane (OMM) and inner mitochondrial membrane (IMM). The IMM serves to enclose the lumen (matrix) of the mitochondria and comprises an inner membrane adjacent to the OMM and a highly convoluted and polymorphic invagination known as the crista. The cristae increase the surface area of the inner membrane while concealing components essential for mitochondrial respiration. The OMM functions as a permeable platform facilitating the convergence of cellular signals, which can be then interpreted and transmitted to the mitochondria. Furthermore, it interfaces with other organelles, such as the ER, lysosomes, and melanosomes, establishing membrane contacts [6].

**Fig. 1:**
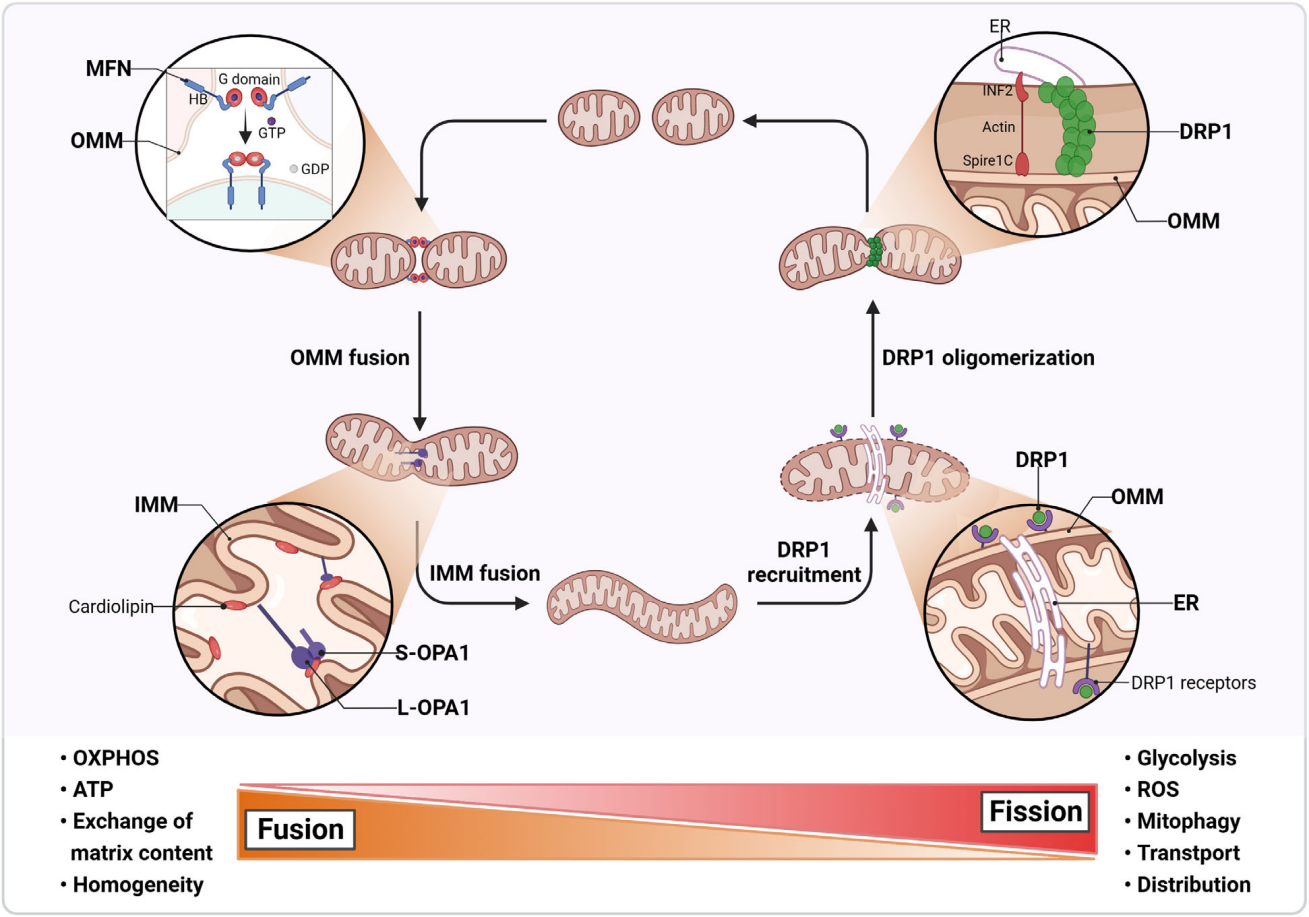
Mechanisms of mitochondrial fusion‐fission dynamics. Mitochondrial fusion involves both outer and inner membrane fusion, facilitating effective OXPHOS, ATP production, and exchange of matrix content (Left). Mitochondrial fission involves ER interactions, DRP1 recruitment and constriction. This process promotes glycolysis, ROS production, mitophagy, and equitable mitochondrial inheritance (Right). ER, endoplasmic reticulum; IMM, inner mitochondrial membrane; OMM, outer mitochondrial membrane; OXPHOS, oxidative phosphorylation; ROS, reactive oxygen species. This figure was created with Biorender.com.

Typical mitochondrial fusion involves the merging of two mitochondria through an end‐to‐end collision, with the membrane fusion event occurring at the collision site [5]. Fusion is a two‐step process: In eukaryotic cells, fusion of the outer membrane is mediated by mitofusin (MFN), whereas that of the inner membrane is mediated by optic atrophy 1 (OPA1) [10]. Both types of dynamin‐like proteins contain a GTPase (G) structural domain and a helical region [11]. Fusion commences with the docking of two trans‐MFN molecules, which may occur through the G structural domain, helix bundle 1 (HB1), or helix bundle 2 (HB2). This binding induces a conformational change in MFN, which prompts the hydrolysis of GTP, ultimately leading to the tethering of the two OMMs [6, 11]. Fusion is most efficient when MFN1 and MFN2 are present simultaneously [12]. The mechanism of IMM fusion is more complex than that of OMM fusion. In IMM, long OPA1 (L‐OPA1) interacts with the trans lipid cardiolipin, and its transient “head‐to‐tail” assembly causes membrane bending, forming unstable tips on two opposing IMMs. When these unstable tips meet, lipid mixing ensues, enlarging the fusion pore and thus completing IMM fusion. Short OPA1 (S‐OPA1), produced by hydrolytic degradation of L‐OPA1, can also interact with liposomes, inducing local membrane bending and giving rise to tubulin‐like structures that facilitate IMM fusion [11, 13]. When four lipid bilayers merge, their contents mix, and matrix components diffuse to form a single, fused mitochondrion [14] (Fig. 1). The deletion of OPA1 leads to a failure in inner membrane fusion, reduced cristae biogenesis, and mitochondrial DNA (mtDNA) instability [15, 16]. The intermediate products of this unsuccessful fusion are eventually degraded through division, causing mitochondrial fragmentation [17]. In addition to typical fusion events, mitochondrial fusion can occur through a transient “kiss‐and‐run” pattern, which does not involve apparent mitochondrial integration or structural rearrangement [18].

### 2.2 Mechanisms of mitochondrial fission

Mitochondrial fission, which typically precedes cell division and mitophagy, represents an essential process for maintaining mitochondrial morphology and altering its function [10]. GTPase dynamin‐related protein 1 (DRP1) is the major mitochondrial fission protein and comprises an N‐terminal GTPase structural domain and a C‐terminal GTPase effector structural domain. Its activity is predominantly regulated by phosphorylation [13, 19]. In response to specific cellular signals, DRP1 translocates from the cytoplasm to the mitochondrial fission site and oligomerizes into a ring‐like structure that mediates the fission of the OMM [20, 21]. The recruitment of DRP1 to the mitochondria depends on several key receptors and recruitment factors, including MFF, MiD49, MiD51, and FIS1, on the outer membrane [10, 14, 22]. Once recruited to the outer membrane, GTP hydrolysis promotes DRP1 helix contraction, leading to the contraction and rupture of the mitochondrial membrane [23]. Although DRP1‐mediated contraction of mitochondrial tubules can be observed, it is insufficient to complete mitochondrial division. Research has revealed that dynamin 2 and Golgi‐derived PI(4)P‐containing vesicles are essential in the final steps of mitochondrial division [24, 25, 26] (Fig. 1).

Mitochondrial contraction and fission take place at ER contact sites, and the initial step in the mitochondrial fission process may involve ER wrapping [27, 28]. This process comprises three crucial steps: (a) INF2, located in the ER, facilitates the distribution of actin to the site of mitochondrial contraction, (b) the recruitment of actin leads to an increase in mitochondrial Ca^2+^ levels, causing the contraction of the inner membrane, and (c) nucleation and polymerization of actin from INF2 in the ER membrane to Spire1C in the OMM promote actin assembly on the mitochondrial surface [13, 29]. The final step of fission depends on the recruitment of DRP1 by MFF, FIS1, and MiDs and its subsequent polymerization. These findings suggest that the ER and cytoskeleton act synergistically to precontract mitochondria and promote the self‐assembly of DRP1 into a multimeric structure, which ultimately cleaves the outer membrane. Furthermore, whether DRP1‐mediated OMM contraction alone is sufficient to simultaneously drive IMM rupture remains unclear. Two inner membrane proteins, S‐OPA1 and MTP18, are thought to play crucial roles in IMM fission [20].

### 2.3 Fusion–fission dynamics and mitochondrial function

While the mechanisms of fusion and fission are distinct, they work in tandem to ensure efficient transport, fair mitochondrial inheritance, and effective oxidative phosphorylation (OXPHOS). A major defect in either of these processes can lead to mitochondrial dysfunction. Mitochondrial morphology is intricately linked to its functions, including energy metabolism, programmed cell death regulation, calcium homeostasis, and ATP and reactive oxygen species (ROS) production [27, 30]. In each cell, a highly regulated balance between fusion and fission events maintains proper mitochondrial morphology, number, and function, which is essential for facilitating the efficient exchange of mitochondrial contents and adapting to changes in cellular metabolic demands [13, 30, 31]. A key role of fusion is to combine the cellular state with mitochondrial function, facilitating communication between mitochondria and host cells. Particularly, mitochondrial fusion promotes OXPHOS, increases ATP production, and enables redistribution of mtDNA between damaged and healthy mitochondria [32]. In contrast, the inhibition or promotion of fission is associated with increased ROS production [33]. The inhibition of MFN1/2 fusion activity induces a mild increase in OMM permeability and caspase 3/7 activation, leading to DNA damage and cell death [12]. Furthermore, mitochondrial fission classifies mitochondria with irreversible mtDNA damage as candidates for autophagy. This asymmetric distribution maintains mitochondrial and cellular homeostasis [13].

Accumulating evidence indicates that cancer cells can disrupt mitochondrial dynamic homeostasis to gain a proliferative and survival advantage. Increased mitochondrial fission has been reported in various cancer types, including human melanoma, CRC, liver cancer, breast cancer, and ovarian cancer [5, 32, 34, 35]. Key effector proteins mediating fission/fusion represent a novel class of drug targets. Recently, the structure–function relationships of these key mitochondrial dynamic proteins have been elucidated, offering valuable insights into their regulation [31]. Several rational drug design and chemical screening activities are currently underway. The discovery of these pharmacological modulators demonstrates the potential of targeting mitochondrial dynamic proteins for cancer treatment [31]. Existing studies have made significant progress in understanding the impact of mitochondrial dynamics on cancer cellular processes. In the subsequent sections, we will delve into the role of mitochondrial dynamics in CRC development.

## 3 Altered mitochondrial fusion–fission dynamics in CRC

Mitochondrial fission and fusion are common events in CRC cells, and an imbalance in these dynamics can either promote or inhibit the development of CRC. To gain a deeper understanding of this relationship, we present a comprehensive overview of the key factors associated with mitochondrial fission and fusion and the pharmacological modulators developed to date. Table 1 provides a summary of the connection between mitochondrial fusion–fission dynamics and CRC.

**Table 1:** Role of mitochondrial dynamics in CRC. AIK, apoptosis inducer kit; CAC, colitis‐associated cancer; CRC, colorectal cancer; DRP1, dynamin‐related protein 1; ER, endoplasmic reticulum; ETC, electron transport chain; FAs, fatty acids; MFF, mitochondrial fission factor; MFN, mitofusin; MMP, mitochondrial membrane potential; mPTP, mitochondrial permeability transition pore; OPA1, optic atrophy 1; OPWPs, oil production waste products; OXPHOS, oxidative phosphorylation; ROS, reactive oxygen species; TCA, tricarboxylic acid cycle.

Molecules/drugs		Models	Effect	Upregulate	Downregulate	Refs
**Fission promotes tumor progression**						
Molecules	DRP1	HCT116, SW480 CDX model	Activation of DRP1 promotes FAs oxidation, induces metabolic reprogramming in CRC cells, and enhances the Wnt/β‐catenin pathway	p‐DRP1^S616^, MFF, p‐ERK, Ki67, LC3II, β‐catenin		[36]
	BRAF^V600E^	Colo‐205, HT‐29, SW480	Mitochondrial fission reprograms glucose metabolism to promote growth, invasion, and migration by activating MEK/ERK signaling downstream of BRAF^V600E^ in CRC cells	DRP1, vimentin, N‐cadherin, PDK1	E‐cadherin	[37]
	RAS^G12V^	HT‐29	RAS^G12V^ promotes rapid mitochondrial fission, potentially through the induction of DRP1 due to an oncogenic MAPK signal	DRP1, ROS	MMP, OCR	[38]
	HMGB1/RAGE	SW480, SW620, LoVo PDX model human CRC tissues	ERK‐mediated high phosphorylation of DRP1 triggers autophagy via the RAGE/ERK pathway, ultimately resulting in chemoresistance	DRP1, HMGB1, ERK1/2, LC3II, RAGE	p62	[40]
	DRP1	HCT116, SW480	Mitochondrial fission factor DRP1 inhibits CRC cell apoptosis	DRP1, MMP	Cytochrome *c*	[41]
	MTFR2	HCT116, Caco‐2, SW480 human CRC tissues	Mitochondrial fission induces the proliferation, invasion, and migration ability of CRC cells	MTFR2, HOXC10, MMP2/9		[42]
	OTUD6A	HCT116, DLD1 CDX model	The overexpression of OTUD6A increases DRP1 levels, induces mitochondrial fragmentation, and enhances CRC cell proliferation	DRP1		[43]
	ARF1‐IQGAP1	DLD1, HCT116, HT29 CDX model Human CRC tissues	ARF1 enhances the interaction of IQGAP1 with ERK and MEK and promotes mitochondrial fission, leading to colon tumorigenesis	DRP1, ARF1, p‐ERK		[44]
	DRP1	LoVo cells	Mitochondrial fission facilitates doxorubicin‐resistant colon cancer cells	DRP1, PON2	OPA1, ROS, TXNIP, SOD2	[45]
	miR‐17‐5p	Human CRC cell lines CDX model Human CRC tissues	miR‐17‐5p induces mitochondrial fission, causing reduced apoptosis and 5‐FU chemoresistance in CRC	miR‐17‐5p, DRP1, BCL2, LC3B	METTL14, MFN2, BAX, caspase 7, p62	[47]
	miR‐27a	HCT116, HT29 mice	The miR‐27a/FOXJ3 axis regulates mitochondrial dynamics and biogenesis, resulting in shorter, fewer, and punctate mitochondria	miR‐27a, MFF, DRP1	FOXJ3, MFN1, MFN2, OPA1	[48]
Drugs	Mdivi‐1	HCT116	Mdivi‐1 inhibits mitochondrial fission, reduces the oxidative metabolism of CRC cells, and impairs cell proliferation		DRP1	[50]
	Ellagic acid	HCT116	Ellagic acid inhibits cell proliferation and promotes widespread cell death by inhibiting DRP1‐mediated mitochondrial fission protein in HCT116 cells		p‐DRP1^S616^ CDK1, cyclin B, Ki67	[51]
	Paris Saponin II	HT29, HCT116 CDX model	Paris Saponin II inhibits ERK1/2 phosphorylation and DRP1 via activating the NF‐κB pathway, triggering cell apoptosis		p‐DRP1^S616^, cyclin D1, c‐Myc, NF‐κB, p‐ERK	[52]
	Sodium butyrate	HCT116, SW480	Sodium butyrate induces G2/M phase cell cycle arrest and inhibits cell viability by suppressing mitochondrial fission	ROS	DRP1, CDK1, cyclin B1, Bcl2	[53]
	Corosolic acid	HCT116, SW480 AOM/DSS model	Corosolic acid exerts anti‐CRC activity by targeting HER2 and HER3 heterodimerization and inhibiting mitochondrial fission	p‐DRP1^S637^, Bax/Bcl, PKA	p‐DRP1^S616^, CDK1	[54]
	ICG‐001	SW620, LoVo, SW480	ICG‐001 inhibits cell viability by inhibiting the DRP1 Ser616 phosphorylation and activating ER stress response in CRC cells		p‐DRP1^S616^	[55]
	Atractylenolide I	HCT116, SW480 AOM/DSS model	Atractylenolide I suppresses NLRP3 inflammasome activation in CAC by inhibiting mitochondrial fission, leading to apoptosis	Bax, PARP	DRP1, NLRP3, ROS, caspase 1, ASC, IL‐1β	[56]
	Pectin	HT29	Pectin inhibits mitochondrial fission and promotes senescence in HT29 cells	MFN1, MFN2, p53, β‐galactosidase, Bak	DRP1, cyclin B1	[57]
	Azelastine	DLD1, HCT116, HT29 CDX model Human CRC tissues	Azelastine might inhibit ARF1‐mediated mitochondrial fission via the ERK signaling pathway to suppress colon tumorigenesis		DRP1, ARF1, Bclxl, Bcl2, p‐ERK	[44]
	Metformin	AOM/DSS model NCM460	Metformin prevents H_2_O_2_‐induced mitochondrial fission by activating the LKB1/AMPK pathway, thereby inhibiting CAC	LKB1, AMPK, NDUFA9	HIF‐1α, PCNA, Akt, NF‐κB	[58]
**Fission inhibits tumor progression**						
Molecules	SIRT3	SW837, SW480	SIRT3‐mediated fatal mitochondrial fission promotes CRC apoptosis by inhibiting the Akt/PTEN pathway	DRP1, MFF, FIS1, caspase 3/9, ROS	SIRT3, Bcl‐2, PTEN, Akt	[61]
	SIRT1	HCT116, DLD1	SIRT1 inhibition promotes mitochondrial acetylation and division, leading to mitochondrial Ca^2+^ overload and CRC apoptosis	DRP1, FIS1, Ca^2+^, ROS	SIRT1, MFN1, MFN2, MMP	[64]
	Yap	SW837	Yap deficiency promotes excessive mitochondrial fission and HtrA2/Omi release through activation of the JNK/DRP1 pathway, ultimately inducing apoptosis	p‐DRP1^S616^, p‐JNK	p‐DRP1^S637^, Yap	[66]
	LATS2	SW480	LATS2 overexpression can overcome 5‐FU resistance by amplifying JNK‐MIEF1‐related mitochondrial fission	LATS2, MIEF1, ROS, DRP1, MFF, Cyt‐c, caspase 9, p‐JNK, Bax	MFN2, OPA1, MMP, ATP, Bcl2	[67]
	DRP1	human CRC tissues	The deficiency of DRP1 may be associated with colon cancer progression		DRP1	[69]
Drugs	Inauhzin	HCT116, DLD1	Inauhzin promotes mitochondrial acetylation and division, leading to mitochondrial Ca^2+^ overload and apoptosis	DRP1, FIS1, Ca^2+^, ROS	SIRT1, MFN1, MFN2, MMP	[64]
	Lycorine	HCT116, HT29, CT26	Lycorine promotes SIRT1‐dependent acetylation of mitochondrial proteins, which significantly exacerbates oxidative stress and mitochondrial fission in CRC	DRP1, ROS	MFN2, ATP, NADPH, MMP	[65]
	Matrine	SW480	Matrine activates MIEF1‐related mitochondrial fission and inhibits SW480 cell survival via the LATS2‐Hippo pathway	MIEF1, DRP1, FIS1	OPA1, MFN2	[68]
	Tanshinone IIA	SW480, SW837	Tanshinone IIA can induce apoptosis and inhibit CRC cell proliferation via the promotion of mitochondrial fission by activating JNK‐MFF and Mst1‐Hippo signaling	DRP1, FIS1, MFF, Mst1, Bax, ROS, caspase 9	MFN2, Opa1, ATP, MMP, CDK4, Bcl2, cyclin D1	[70, 71]
	*Aloe gel* glucomannan	CT26 cells	*Aloe gel* glucomannan upregulates mitophagy and mitochondrial fission signaling in CT26 cells and drives cancer cell death	LC3II, ROS, DRP1, AMPK, PINK1, Parkin	p62	[72]
	Camptothecin, Triptolide, AIK	DLD1, HCT‐116	Camptothecin, triptolide, and AIK target mitochondria through the process of fission and affect the induction of apoptosis in CRC cells	p‐DRP1^S616^/p‐DRP1^S637^, ROS	MMP, ATP, OXPHOS	[73]
	YQ456	HCT116, LoVo, SW480 PDX model	YQ456 can dephosphorylate DRP1 at S637 in CRC cells, resulting in sustained mitochondrial fission and cell death	DRP1, ROS, p62	MMP, PINK1, Parkin, Lc3II	[74]
**Fusion promotes tumor progression**						
Molecules	CHD6‐ TMEM65	DLD1, HCT116, SW620 AOM/DSS model PDX/CDX model Human CRC tissues	CHD6 induces TMEM65‐mitochondrial fusion, increases ATP production, and promotes CRC progression via EGF and Wnt/β‐catenin signaling	CHD6, TMEM65, EGF, Opa1, OCR, β‐catenin, ATP, TFAM, p‐AKT	DRP1, GSK3β, FBXW7, ROS, Parkin	[75]
Drugs	RKI‐1447	HCT116, SW480, DLD1 CDX model	RKI‐1447 treatment promotes ER stress‐related apoptosis by inhibiting mitochondrial fusion in CRC cells	DRP1, ROS, p‐eIF2α, CHOP, cleaved‐OMA1	OPA1, OCR, ATP, MMP	[77]
	*Paris polyphylla*	Caco‐2, HT‐29	Active ingredient isolated from *P. polyphylla* inhibits *Fusobacterium nucleatum*‐induced mitochondrial fusion and cell migration		OPA1	[78]
**Fusion inhibits tumor progression**						
Molecules	MCCC2	HCT116 Human CRC tissues	*MCCC2* knockdown induces mitochondrial fusion, thereby inhibiting CRC cell proliferation, invasion, and migration	MFN1, MFN2, OPA1	MCCC2	[79]
	IGF1R	HT‐29 AOM/DSS model	Heterozygous knockdown of IGF‐1R protects colonic epithelial cells from oxidative stress and prevents colon tumorigenesis by activating mitochondrial fusion function	MFN1, MFN2, OPA1, GSK3β, E‐cadherin	IGF‐1R, p‐ERK, cyclin D1, β‐catenin	[80]
	OPWPs Hydroxytyrosol	HCT116, LoVo	OPWP extracts and hydroxytyrosol promote mitochondrial fusion via the PPARγ/PGC‐1α axis, causing proliferation inhibition and apoptosis in CRC cells	MFN1/2, ATP, MMP, TOM20, FOXJ3, PGC‐1α, OXPHOS	MFF	[81]
Drugs	2‐DG	DLD1	The 2‐DG treatment induces activation of AMPK, leading to increased mitochondrial fusion and decreased fission, ultimately inhibiting glycolysis in cancer cells	MFN1, MFN2, MMP, AMPK	DRP1, ATP	[82]

### 3.1 Fission promotes CRC progression

#### 3.1.1 Molecules associated with fission

**Fig. 2:**
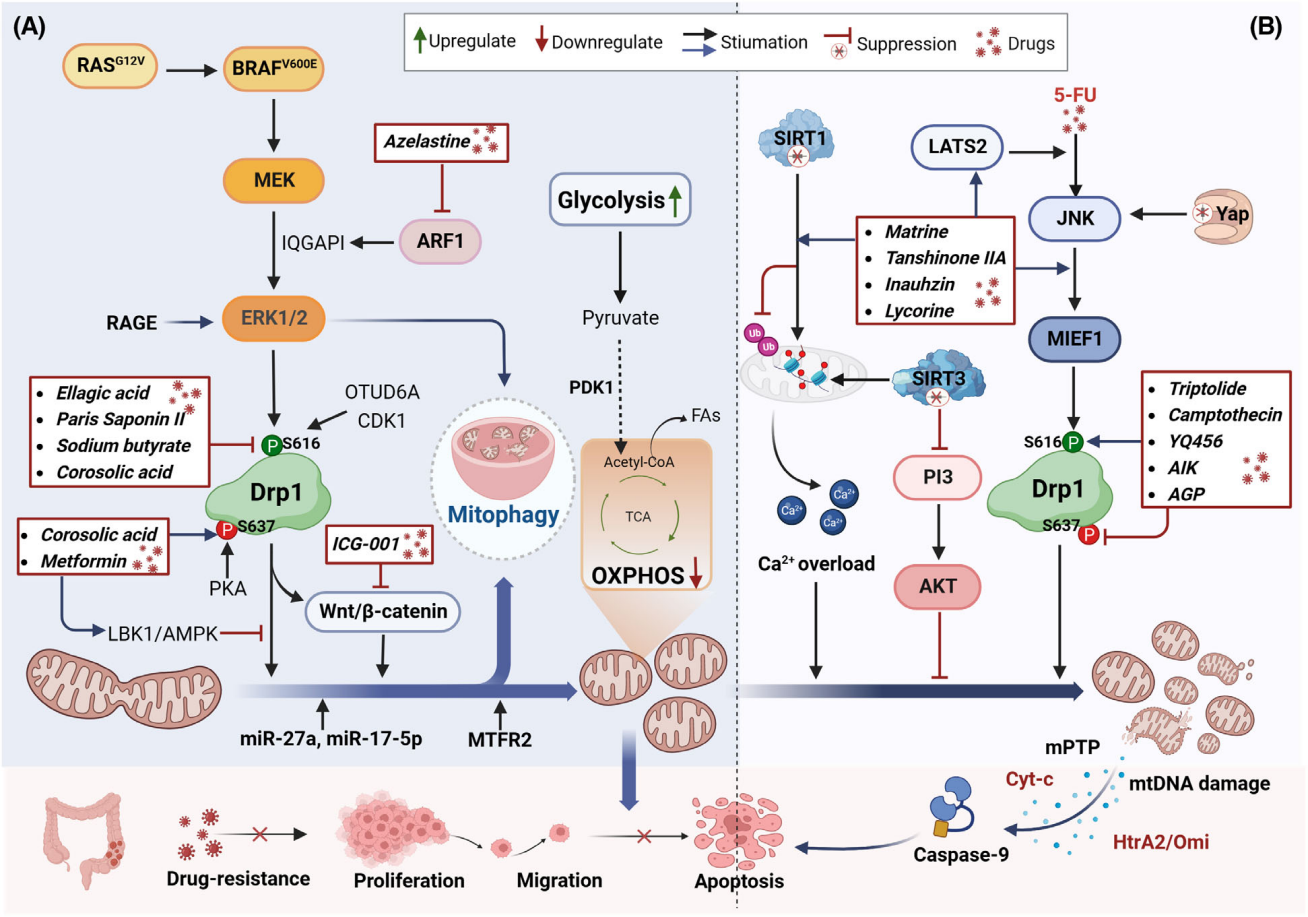
Role of mitochondrial fission in the progression of colorectal cancer. (A) Mitochondrial fission, facilitated by activated DRP1S616, contributes to tumor progression by driving metabolic reprogramming and enhancing cell proliferation, migration, and drug resistance. (B) Excessive mitochondrial fission, stemming from the deficiency or overexpression of key genes, triggers mitochondria‐mediated apoptosis, leading to the inhibition of tumor progression. FAs, fatty acids; mPTP, mitochondrial permeability transition pore; OXPHOS, oxidative phosphorylation; TCA, tricarboxylic acid cycle. This figure was created with Biorender.com.

DRP1 is a crucial effector protein in mitochondrial fission. DRP1‐mediated mitochondrial fission plays a crucial role in regulating FAs and glucose metabolism, which, in turn, promotes CRC cell proliferation, invasion, and migration [36, 37]. Mechanistically, the uptake of FAs activates the phosphorylation of DRP1 at the Ser616 site and enhances the interaction between DRP1 and MFF, thereby inducing mitochondrial fission in HCT116 cells. This activation of DRP1^S616^ promotes FAs oxidation‐dependent acetylation of β‐catenin, which enhances Wnt/β‐catenin signaling [36]. At the level of glucose metabolism, *BRAF* ^V600E^ CRC cells activate MEK/ERK signaling, followed by activation of p‐DRP1^S616^. This leads to increased mitochondrial fragmentation, resulting in tumor cells exhibiting a glycolytic phenotype and enhanced metastatic dominance [37, 38]. This glycolytic phenotype is partially dependent on mitochondrial pyruvate dehydrogenase kinase 1 (PDK1), which positively influences mitochondrial fragmentation, implicating PDK1 as a potential target for intervention in mitochondrial fission [37, 39]. No significant changes have been observed in the levels of total DRP1 or other mitochondrial dynamic‐related proteins such as MFN1, MFN12, and OPA1 [36, 37]. Furthermore, in the *BRAF* ^V600E^ HT‐29 cell line, the deletion of DRP1 prevents RAS^G12V^‐induced mitochondrial division, and the DRP1^S616^ phosphorylation status is sufficient to characterize transformation‐induced mitochondrial dysfunction. This can distinguish *BRAF* ^V600E^‐positive lesions from *BRAF* ^Wt^ [38]. These findings suggest that CRC driven by mutations in *BRAF* and *RAS* is characterized by mitochondrial fragmentation. The phosphorylation status of DRP1^S616^ and the degree of mitochondrial fission may serve as useful clinical biomarkers for confirming the *BRAF* ^V600E^ status and, potentially, any tumor with oncogenic MAPK pathway mutations [37, 38]. Combining small molecule inhibitors of these mutations with inhibitors of mitochondrial fission may have an unanticipated role in enhancing chemotherapeutic success. Other studies have demonstrated that ERK‐triggered high DRP1^S616^ phosphorylation can activate autophagy through the RAGE/ERK1/2 pathway, resulting in chemoresistance and cancer cell regeneration [40] (Fig. 2A). In contrast, reduced DRP1 expression leads to mitochondria elongation, decreased mitochondrial membrane potential (MMP), cytochrome *c* release, and apoptosis in CRC cells [41].

In addition to DRP1, other key genes/proteins mediating mitochondrial fission have been identified in CRC. Mitochondrial fission regulator 2 (*MTFR2*), *OTUD6A*, and ARF1 are overexpressed in CRC tissues [42, 43, 44]. Knocking down *MTFR2* in HCT116 cells significantly decreased cancer cell proliferation, invasion, and migration abilities [42]. *OTUD6A* stabilizes DRP1 through deubiquitylation, and its aberrant expression in CRC cells prolongs the half‐life of DRP1, leading to mitochondrial fragmentation and promoting cell proliferation and colony formation [43]. ARF1 has been found to activate ERK signaling by enhancing the interaction of IQGAP1 with ERK and MEK, thus promoting mitochondrial division and colon tumorigenesis [44]. Chemoresistance presents a major challenge in cancer treatment. Research has established that doxorubicin‐ or oxaliplatin‐resistant colon cancer cells exhibit pronounced mitochondrial fragmentation due to increased division [40, 45]. Although studies on mitochondrial morphology in the context of drug resistance in CRC stem cells (CCSCs) are lacking, the elevated mitophagy rate implies that mitochondrial fission is a mechanism underlying chemotherapy resistance in CCSCs [46]. miR‐17‐5p and miR‐27a induce mitochondrial fission, leading to reduced apoptosis and 5‐FU chemoresistance in CRC [47, 48]. Mechanistically, Mettl14‐dependent pri‐miR‐17 maturation can directly bind to MFN2, resulting in reduced mitochondrial fusion, enhanced mitochondrial division, and autophagy [47]. Similarly, when miR‐27a was knocked down in HCT116 cells, fusion proteins were upregulated, whereas fission proteins were downregulated and mitochondrial abundance was increased [48] (Fig. 2A). miRNAs and their corresponding target genes are involved in the regulation of mitochondrial dynamics, metabolic reprogramming, and chemotherapy resistance [49]. These studies further support the role of miRNAs in regulating mitochondrial function and homeostasis, highlighting their potential as predictive molecules for CRC treatment response [47, 48].

#### 3.1.2 Fission inhibitors

In addition to the aforementioned research on key genes and proteins, multiple studies investigating the anticancer activity of naturally occurring compounds and specific drugs have confirmed that mitochondrial fission promotes CRC progression. For instance, Mdivi‐1, a commonly used inhibitor of DRP1, can inhibit mitochondrial fission, reduce oxidative metabolism in CRC cells, and impede cell proliferation [50]. When human CRC cell lines were exposed to ellagic acid [51], Paris Saponin II [52], sodium butyrate [53], corosolic acid [54], and the Wnt/β‐catenin signaling inhibitor ICG‐001 [55], the initially small and punctate mitochondrial morphology transitioned to a state of hyperperfusion. This change resulted in reduced MMP, impaired mitochondrial respiration, and limited cell proliferation. Mechanistically, these compounds dephosphorylate DRP1 at the Ser616 site, thereby inhibiting mitochondrial fission and downregulating cell proliferation and cell cycle markers [51, 52, 53, 54, 55]. Additionally, Paris Saponin II inhibits DRP1 recruitment by activating the NF‐κB pathway, leading to cell cycle arrest and apoptosis [52]. Corosolic acid prevents mitochondrial fission by decreasing CDK1 expression, increasing PKA expression, inhibiting HER2/HER3 heterodimerization, and regulating the phosphorylation of DRP1 at the Ser616 (CDK1‐dependent) and Ser637 (PKA‐dependent) sites [54]. Although corosolic acid does not significantly affect OPA1 and MFN1/2 expression, it is expected to be a novel HER2/HER3 heterodimerization inhibitor against CRC [54]. ICG‐001 exerts antiproliferative activity by activating the early ER stress response [55]. Atractylenolide I, the major bioactive component in *Atractylodes macrocephala*, blocks DRP1‐mediated mitochondrial fission, inhibits the activation of NLRP3 inflammasome in colitis‐associated cancer (CAC), and disrupts mitochondrial membrane integrity, thereby inducing apoptosis [56]. Pectin, a polysaccharide, has been found to decrease DRP1 expression and increase mitochondrial fusion‐ and cellular senescence‐associated protein expression (e.g., β‐galactosidase and p53), thereby reducing cell viability and promoting cellular senescence [57]. Some commonly used drugs for the treatment of chronic diseases have also been reported to inhibit CRC development, possibly by modulating mitochondrial dynamics. For example, the antiallergic drug azelastine can inhibit ARF1‐mediated mitochondrial fission through activation of ERK signaling [44]. The glucose‐lowering drug metformin may protect the mitochondrial structure of colonic epithelial cells by activating the *LKB1*/AMPK pathway and inhibiting H_2_O_2_‐induced mitochondrial fission, thereby delaying the progression of CAC [58] (Fig. 2A).

Collectively, enhanced mitochondrial fission in CRC is often a result of aberrantly expressed genes. At the molecular level, mitochondrial fission is primarily induced by ERK1/2‐triggered phosphorylation of DRP1^S616^ and dephosphorylation of DRP1^S637^ in cancer cells. This enables cancer cells to undergo metabolic reprogramming and gain advantages in proliferation, invasion, migration, and chemoresistance. Exploiting this mechanism, some compounds inhibit mitochondrial fission by inhibiting the oncogenic MAPK pathway, dephosphorylating DRP1^S616^, and blocking the recruitment of DRP1, thereby decreasing the viability of cancer cells (Fig. 2A). These findings suggest that targeting mitochondrial fission may contribute to the development of small molecule inhibitors of the oncogenic MAPK pathway.

### 3.2 Fission inhibits CRC progression

#### 3.2.1 Molecules associated with excessive fission

Normal mitochondrial fission promotes efficient energy metabolism. However, excessive fission is believed to have a negative impact on the viability of CRC cells. SIRT3 and SIRT1 are deacetylases located in mitochondria that promote CRC proliferation and metastasis [59, 60]. SIRT3 regulates CRC stress response by modulating mitochondrial fission, and SIRT3 deficiency triggers lethal mitochondrial fission through the inhibition of the Akt/PTEN pathway [61]. Abnormal mitochondrial fission disrupts mtDNA replication and transcription, causing mitochondrial dysfunction and energy shortage in tumor cells [61, 62]. Damaged mitochondria open the mitochondrial permeability transition pore (mPTP), releasing cytochrome *c* into the cytoplasm/nucleus and inducing caspase 9‐related mitochondrial apoptosis [61, 63]. Similarly, the mitochondrial morphology of SIRT1‐deficient CRC cells changes significantly from tubular to punctate, accompanied by corresponding changes in the levels of fission and fusion indices [64, 65]. Mechanistically, inhibition of SIRT1 enhances protein acetylation in mitochondria but prevents mitochondrial ubiquitination and mitophagy degradation, leading to mitochondrial Ca^2+^ overload and depolarization. Under cellular stress, Ca^2+^ overload induces MMP reduction and ROS burst, ultimately promoting apoptosis [64, 65]. These findings reveal the crucial role of SIRTs in regulating mitochondrial dynamics and function, suggesting a possible pathway for anticancer intervention through SIRT3 and SIRT1 inhibition (Fig. 2B).

In addition to SIRTs, Yap deficiency can induce high levels of mitochondrial fission by activating JNK/DRP1^S616^ signaling, leading to the leakage of HtrA2/Omi into the cytoplasm and the eventual activation of the mitochondrial apoptotic pathway [66]. Reduced LATS2 expression represents one of the mechanisms of 5‐FU chemoresistance, and when combined with 5‐FU, LATS2 overexpression enhances the efficacy of chemotherapy [67]. Mechanistically, LATS2 overexpression further amplifies 5‐FU‐induced JNK‐MIEF1‐associated mitochondrial fission, enhances the release of pro‐apoptotic factors into the nucleus, and ultimately upregulates Bax‐related mitochondrial apoptosis [67]. Consistent with this study, the natural compound matrine has been proven to activate MIEF1‐related mitochondrial fission and promote apoptosis in SW480 cells through the LATS2‐Hippo pathway [68] (Fig. 2B).

Although many studies have reported elevated DRP1 expression in CRC, its precise contribution to the development of CRC remains unclear. To explore the role of DRP1 in colon cancer, Kim et al. [69] examined DRP1 levels in human colon cancer tissues. They found that DRP1 levels were lower in 75% of colon cancer tissues than in adjacent normal tissues, and DRP1 was upregulated in 25% of tumor tissues. Furthermore, they observed that DRP1 levels decreased particularly in mid‐ to late‐stage colon cancer, suggesting a possible association between a lack of DRP1 or a deficiency in mitochondrial division and colon cancer progression. Additionally, the rate of decline was found to be higher in male patients than in female patients [69]. These findings differ from previous research and reveal that the effects of changes in DRP1 levels may vary depending on the cell type, tissue, or physiological context.

#### 3.2.2 Fission promoters

Several compounds that induce mitochondrial fission in CRC cells have been developed and include inauhzin [64], lycorine [65], matrine [68], tanshinone IIA [70, 71], and *Aloe gel* glucomannan [72]. As mentioned previously, inauhzin and lycorine primarily promote SIRT1‐dependent acetylation of mitochondrial proteins, significantly exacerbating oxidative stress and mitochondrial fission in CRC cells [64, 65]. IDH1 has been identified as a key cellular target of lycorine, showing promise as a potential therapeutic target for CRC [65]. Matrine and tanshinone IIA have been shown to induce apoptosis in CRC cells by promoting mitochondrial fission through the LATS2‐Hipp [68], Mst1‐Hippo [70], and JNK‐Mff [71] pathways. *Aloe gel* glucomannan upregulates mitophagy and mitochondrial fission signaling in CT26 cells, inducing mitochondrial damage and ROS overproduction, thus driving cancer cell death [72]. In addition to the aforementioned small molecules of natural origin, the chemotherapeutic drugs triptolide and camptothecin, myoferlin inhibitor YQ456, and apoptosis inducer kit (AIK) stimulate DRP1^S616^ phosphorylation and DRP1^S637^ dephosphorylation. This dual action results in sustained mitochondrial fission, ultimately leading to cell death [73, 74] (Fig. 2B). These studies illustrate that activation of sustained mitochondrial division promotes apoptosis in CRC cells, and these natural compounds developed on this basis show promising anticancer activities and clinical applications. However, most of the current studies are limited to the molecular level and remain controversial. Further in‐depth mechanistic investigations and gradual validation in clinical practice are needed in the future.

Collectively, mitochondrial fission appears to play a dual role in CRC progression, both promoting and inhibiting it. DRP1^S616^ phosphorylation promotes mitochondrial fission and metabolic reprogramming, inducing CRC cell proliferation, invasion, migration, and chemoresistance. However, excessive fission due to the deficiency or overexpression of certain genes triggers mitochondria‐mediated apoptosis (Fig. 2). In conclusion, mitochondrial fission enhances CRC cell growth viability, whereas excessive fission induces apoptosis. These findings provide the theoretical basis for finding the optimal time to intervene in CRC by targeting mitochondrial fission.

### 3.3 Fusion promotes CRC progression

**Fig. 3:**
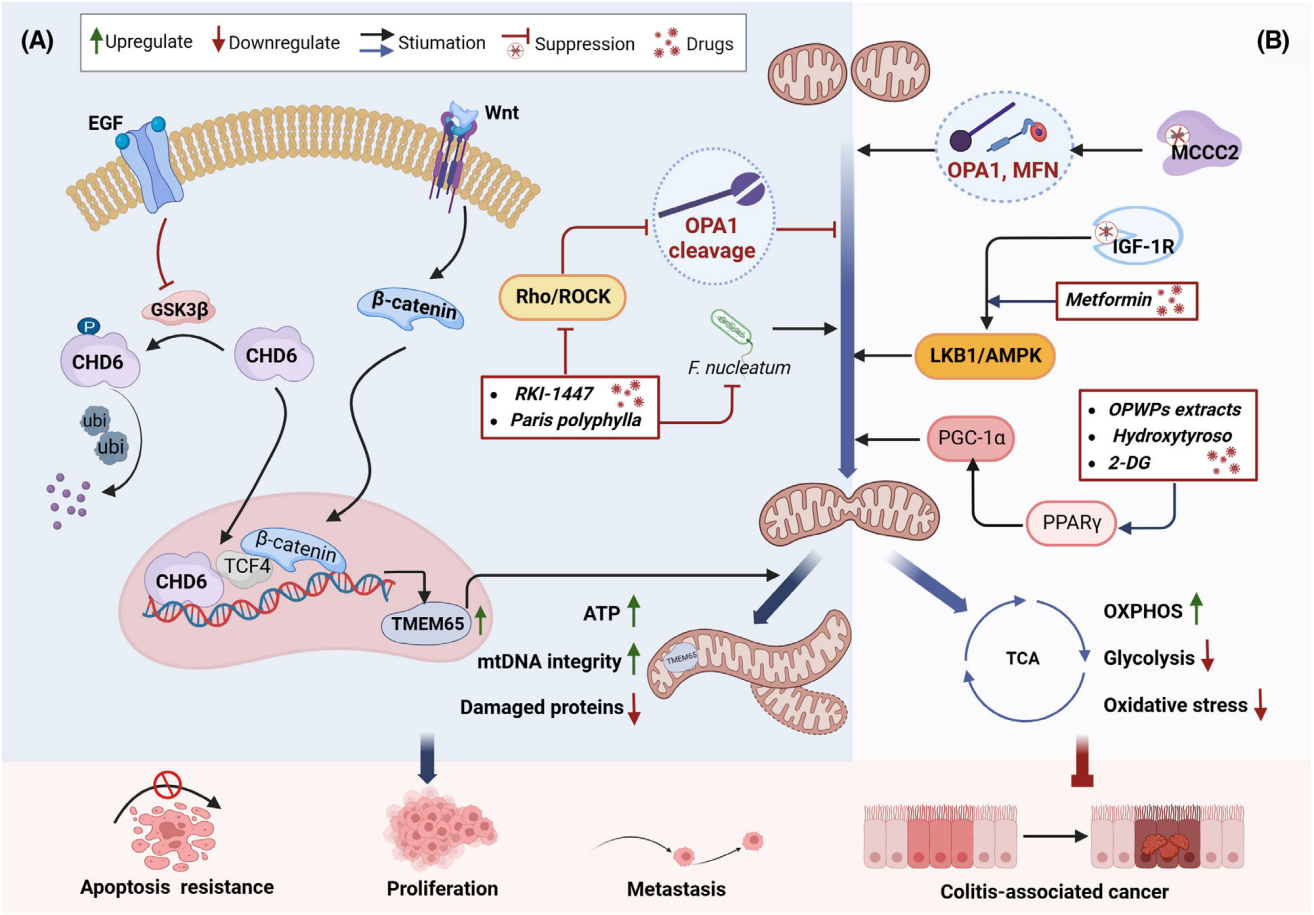
Role of mitochondrial fusion in the progression of colorectal cancer. (A) Excessive mitochondrial fusion promotes cell proliferation, metastasis, and apoptosis resistance. (B) Mitochondrial fusion promotes OXPHOS and inhibits glycolysis. It also protects intestinal epithelial cells from oxidative stress. OXPHOS, oxidative phosphorylation; TCA, tricarboxylic acid cycle. This figure was created with Biorender.com.

Mitochondrial fusion, in contrast to mitochondrial fission, plays a key role in the progression of CRC. Recent studies have revealed that overexpression of the chromodomain helicase DNA binding protein 6 (*CHD6*) gene in CRC promotes excessive mitochondrial fusion and cell proliferation. Mechanistically, aberrant activation of the upstream EGF‐GSK3β signaling pathway hinders the ubiquitination and degradation of CHD6. This leads to the accumulation of structurally stable CHD6 in the nucleus, where it enhances the transcription of the IMM protein TMEM65 through the Wnt/β‐catenin signaling pathway. This process results in induced sustained mitochondrial fusion, ATP overproduction, tumor growth, and metastasis. In contrast, *CHD6* knockdown in CRC cells reduces the length of mitochondria and the number of cristae, ultimately leading to apoptosis [75]. Mitochondrial fusion not only promotes cancer cell proliferation but also repairs mitochondria and maintains mtDNA integrity by diluting damaged proteins under conditions of oxidative stress [76] (Fig. 3A).

Currently, some small molecule compounds, such as RKI‐1447 [77] and *Paris polyphylla* [78], have shown promise in inhibiting mitochondrial fusion and exerting anti‐CRC activity. RKI‐1447, a novel Rho/ROCK inhibitor, induces mitochondrial depolarization that activates OPA1 cleavage and triggers ER stress. This leads to impaired mitochondrial fusion and mitochondrial respiration repression, ultimately resulting in ER stress‐related apoptosis [77]. Active ingredients isolated from *P. polyphylla* inhibit mitochondrial fusion and migration induced by the pathogenic bacterium *Fusobacterium nucleatum* in Caco‐2 cells [78] (Fig. 3A). These studies suggest that herbal medicines and their active ingredients targeting mitochondrial fusion may be potential candidates for complementary chemotherapy in CRC.

### 3.4 Fusion inhibits CRC progression

Although excessive mitochondrial fusion can promote CRC progression, the deletion of certain oncogenes can inhibit tumor growth. For instance, the mitochondrial gene *MCCC2* serves as a mediator between mitochondria and telomeres. *MCCC2* knockdown upregulates fusion markers, MFN1/2 and OPA1, to induce mitochondrial fusion, thereby inhibiting CRC cell proliferation, invasion, and migration [79]. IGF‐1R, a major determinant of cancer, exhibits protective effects against oxidative stress damage when subjected to heterozygous knockdown in colonic epithelial cells. This protective action is achieved through activation of the *LKB1*/AMPK pathway by enhancing mitochondrial fusion functions to maintain mitochondrial structural integrity. Additionally, this process plays a preventive role in AOM/DSS‐induced CAC [80] (Fig. 3B).

Specific compounds, such as oil production waste product (OPWP) extracts, hydroxytyrosol [81], and the glucose analog 2‐DG [82], may intervene in colonic tumor growth by promoting mitochondrial fusion into network structures. Mechanistically, OPWP extracts and hydroxytyrosol promote mitochondrial fusion through the PPARγ/PGC‐1α axis and upregulate OXPHOS signaling, ultimately inhibiting cell proliferation and inducing apoptosis [81]. Tumor cells are known to primarily rely on glycolysis for energy production. Recent research has shown that when colon cancer cells are exposed to the glucose analog 2‐DG, fusion and fission proteins become imbalanced, and mitochondria fuse into complex network structures. This phenomenon is accompanied by a marked inhibition of glycolysis [37, 82], although it does not affect the viability of colon cancer cells [82] (Fig. 3B).

In summary, excessive mitochondrial fusion can generate sufficient energy and repair damaged mitochondria. It can also protect intestinal epithelial cells from oxidative stress damage and resist aberrant mitochondrial division, thus preventing CACs. Additionally, mitochondrial fusion partially inhibits the glycolytic pathway, thereby slowing down CRC progression (Fig. 3). These findings suggest that targeting mitochondrial fusion could be a new therapeutic strategy for CRC treatment. Nevertheless, additional higher quality research is required to fully understand the role of mitochondrial fusion in CRC.

## 4 Mitochondrial dynamics in other cells associated with CRC

**Fig. 4:**
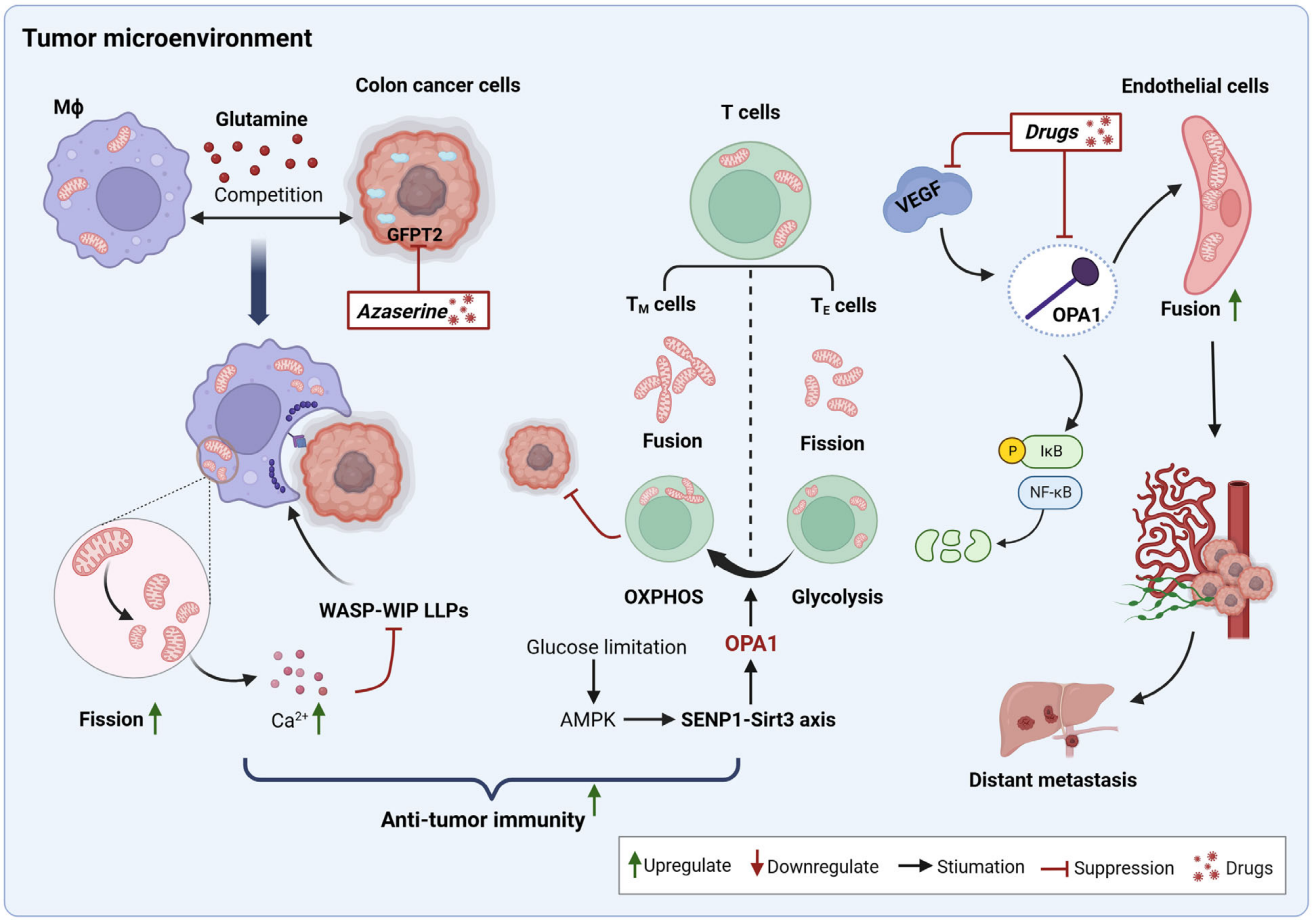
Mitochondrial dynamics in other cells in the tumor microenvironment. Activating mitochondrial fission in macrophages or mitochondrial fusion in T cells improves anti‐tumor immunity. Mitochondrial fusion in vascular endothelial cells promotes tumor angiogenesis and distant metastasis. OXPHOS, oxidative phosphorylation. This figure was created with Biorender.com.

Imbalances in mitochondrial fusion–fission dynamics not only occur within CRC cells but also in immune and endothelial cells in the tumor microenvironment. Recent studies indicate that DRP1‐mediated mitochondrial fission in macrophages plays a crucial role in effectively phagocytosing live tumors and apoptotic cells [83, 84, 85]. Additionally, mitochondrial fission is essential for priming immunity in bone marrow‐derived macrophages, enabling them to regulate tumor metastasis [86]. Specifically, mitochondrial fission in macrophages increases intracytoplasmic calcium ion concentration, disrupting the liquid–liquid phase separation (LLPS) of the WASP–WIP complex in macrophages and promoting actin rearrangement. This ultimately enhances the phagocytic activity of macrophages. Stimulation with therapeutic monoclonal antibodies further induces phagocytic activity in macrophages [83]. Overexpression of GFPT2, a protease competing with macrophages for glutamine, can impair mitochondrial fission in macrophages of colon cancer cells, leading to a weakened phagocytic response and drug resistance [83]. These results indicate that inducing mitochondrial fission in macrophages by targeting GFPT2 may enhance the efficacy of therapeutic monoclonal antibodies. In addition to macrophages, the mitochondrial dynamics in T cells are linked to their antitumor immunity. Mitochondrial fusion in memory T (T_M_) cells favors OXPHOS and FAs oxidation, whereas mitochondrial fission in effector T (T_E_) cells reduces electron transport chain (ETC) efficiency and promotes aerobic glycolysis [87]. Under glucose limitation, the AMPK/SENP1‐Sirt3 axis is activated to promote memory development and T cell survival while inhibiting OPA1 segmentation in T_M_ cells, leading to enhanced mitochondrial fusion in T_M_ cells. This, in turn, inhibits colon tumor growth in MC38‐OVA model mice [88]. A low response rate is a major challenge in the current clinical application of immune checkpoint inhibitors for CRC treatment. These findings suggest that targeting mitochondrial dynamics, such as activating mitochondrial fission in macrophages or mitochondrial fusion in T cells, may help improve the efficacy of immunotherapy in CRC (Fig. 4).

Tumor angiogenesis is a key factor in the rapid growth and metastasis of tumors. Studies indicate that the proangiogenic factor VEGF stimulates the increase of OPA1 protein levels in endothelial cells, leading to prolonged mitochondria and inhibition of the NF‐κB pathway. This ultimately enhances the expression of angiogenic and lymphatic genes, promoting tumor growth and metastasis [89] (Fig. 4). In addition to immune and endothelial cells, altered mitochondrial dynamics were observed in myocytes from chemotherapy‐induced C26 cachexia mice. Unusually, the expression of mitochondrial fusion, fission, and biogenesis markers was reduced in myocytes. These results highlight the potential for targeting mitochondrial dynamics in myocytes or endothelial cells to develop combination therapies that mitigate the side effects of chemotherapy.

## 5 Conclusions and future perspectives

Mitochondria have emerged as a key area of study for understanding the pathogenesis of intestinal tumors. According to our review findings, mitochondrial fission has been extensively studied in CRC progression. Activation of phosphorylation of DRP1^S616^, the most critical protein for mitochondrial fission, is induced by various endogenous and exogenous factors. This activation facilitates the recruitment of DRP1 from the cytoplasm to the mitochondrial surface. Other fission proteins, such as MFF and FIS1, assist DRP1 in triggering mitochondrial fission, which promotes metabolic reprogramming in cancer cells and activates oncogenic signaling pathways. However, excessive mitochondrial fission can stimulate the mitochondrial apoptotic pathway and induce apoptosis in CRC cells. Therefore, to develop effective interventions for CRC, comprehending the various pathological processes occurring at different stages of the disease is necessary. This understanding will enable the development of “inhibitors” or “promoters” that can effectively target mitochondrial fission. Furthermore, research has delved into mitochondrial fusion dysfunction concerning CRC. Sustained mitochondrial fusion leads to an overproduction of ATP, diluting damaged mitochondrial proteins and maintaining the integrity of mtDNA. This creates a favorable environment for cancer cell growth. Moreover, mitochondrial fusion helps counteract abnormal mitochondria division, protects intestinal epithelial cells from oxidative stress, and potentially prevents the early onset of CAC. These studies provide compelling evidence that mitochondrial dynamics significantly regulate the unique metabolism, rapid proliferative capacity, drug resistance, and diverse apoptotic pathways of CRC cells. Any disruption to the balance of fusion and fission, whether excessive or insufficient, can profoundly affect the advancement and treatment of CRC. Consequently, the development of drugs that selectively target mitochondrial fusion–fission dynamics in CRC cells presents a promising avenue. This can be achieved either by restoring fusion–fission homeostasis to inhibit cell proliferation, migration, and drug resistance or by inducing fusion–fission imbalance to promote apoptosis. Furthermore, our review emphasizes that manipulating mitochondrial dynamics in immune and endothelial cells within the tumor microenvironment might enhance the sensitivity of immunotherapy or targeted therapy. Pathological mitochondrial fusion or fission typically results from uncontrolled mitochondrial dynamic proteins, which are regulated by complex molecular mechanisms. A deeper understanding of the structure–function relationships of these dynamic proteins is required to develop effective “inhibitors” or “promoters.” Consistent with our review, a recent study has highlighted mitochondrial dynamic proteins as potential drug targets for the treatment of cancer [31].

To gain a better understanding of the role of mitochondrial fusion–fission dynamics in CRC development, future studies should address several issues. First, whether the disruption of mitochondrial dynamic balance in CRC is a primary or secondary factor in other pathological processes remains unclear. Second, mitochondrial dynamics have multiple physiological effects, and alterations in these dynamics can affect various cellular processes. However, current studies have primarily focused on observing changes in mitochondrial morphology and detecting molecules associated with morphological changes. Determining how mitochondrial dynamics affect pathological processes critical to CRC cell proliferation or apoptosis is challenging. Additionally, further evidence is needed to determine whether the effects of mitochondrial dynamics are specific to certain cell types. Third, further research is required to establish the relationship between mitochondrial morphology and cellular metabolism. Present studies frequently assume that elongated mitochondria are characteristic of OXPHOS cells, whereas small and fragmented mitochondria are typical of glycolytic cells. However, this perspective may oversimplify the matter. Once the interconnections between mitochondrial fusion–fission dynamics and cellular processes, including cell metabolism, proliferation, invasion, migration, apoptosis, and drug resistance, are better understood, targeted therapy focusing on mitochondrial dynamics could be a promising approach for the prevention and treatment of CRC.

## Conflict of interest

The authors declare no conflict of interest.

## Author contributions

ZW designed the review protocol, conducted the search, drafted the manuscript, and prepared all figures and tables. CX, FL, and WH screened potentially eligible studies, extracted and analyzed data, and updated reference lists. CX and FY were responsible for the funding acquisition. XL contributed to the design of the review protocol, arbitrating potentially eligible studies, and interpreting results. FY and XL provided feedback on the report. All authors approved the final version of the manuscript.

## References

[1] Siegel RL , Wagle NS , Cercek A , Smith RA , Jemal A . Colorectal cancer statistics, 2023. CA Cancer J Clin. 2023;73:233–254. 10.3322/caac.21772 36856579

[2] Colloca A , Balestrieri A , Anastasio C , Balestrieri ML , D'Onofrio N . Mitochondrial sirtuins in chronic degenerative diseases: new metabolic targets in colorectal cancer. Int J Mol Sci. 2022;23:3212. 10.3390/ijms23063212 35328633 PMC8949044

[3] Sessions DT , Kashatus DF . Mitochondrial dynamics in cancer stem cells. Cell Mol Life Sci. 2021;78:3803–3816. 10.1007/s00018-021-03773-2 33580834 PMC11071759

[4] Zhou Z , Fan Y , Zong R , Tan K . The mitochondrial unfolded protein response: a multitasking giant in the fight against human diseases. Ageing Res Rev. 2022;81:101702. 10.1016/j.arr.2022.101702 35908669

[5] Chan DC . Mitochondrial dynamics and its involvement in disease. Annu Rev Pathol. 2020;15:235–259. 10.1146/annurev-pathmechdis-012419-032711 31585519

[6] Giacomello M , Pyakurel A , Glytsou C , Scorrano L . The cell biology of mitochondrial membrane dynamics. Nat Rev Mol Cell Biol. 2020;21:204–224. 10.1038/s41580-020-0210-7 32071438

[7] Klos P , Dabravolski SA . The role of mitochondria dysfunction in inflammatory bowel diseases and colorectal cancer. Int J Mol Sci. 2021;22:11673. 10.3390/ijms222111673 34769108 PMC8584106

[8] Tan HWS , Lu G , Dong H , Cho YL , Natalia A , Wang L , et al. A degradative to secretory autophagy switch mediates mitochondria clearance in the absence of the mATG8‐conjugation machinery. Nat Commun. 2022;13:3720. 10.1038/s41467-022-31213-7 35764633 PMC9240011

[9] Kasprzak A . Autophagy and the insulin‐like growth factor (IGF) system in colonic cells: implications for colorectal neoplasia. Int J Mol Sci. 2023;24:3665. 10.3390/ijms24043665 36835075 PMC9959216

[10] Ul Fatima N , Ananthanarayanan V . Mitochondrial movers and shapers: recent insights into regulators of fission, fusion and transport. Curr Opin Cell Biol. 2023;80:102150. 10.1016/j.ceb.2022.102150 36580830

[11] Gao S , Hu JJ . Mitochondrial fusion: the machineries in and out. Trends Cell Biol. 2021;31:62–74. 10.1016/j.tcb.2020.09.008 33092941

[12] Zacharioudakis E , Agianian B , Mv VK , Biris N , Garner TP , Rabinovich‐Nikitin I , et al. Modulating mitofusins to control mitochondrial function and signaling. Nat Commun. 2022;13:3775. 10.1038/s41467-022-31324-1 35798717 PMC9262907

[13] Quintana‐Cabrera R , Scorrano L . Determinants and outcomes of mitochondrial dynamics. Mol Cell. 2023;83:857–876. 10.1016/j.molcel.2023.02.012 36889315

[14] Chen H , Chan DC . Mitochondrial dynamics in regulating the unique phenotypes of cancer and stem cells. Cell Metab. 2017;26:39–48. 10.1016/j.cmet.2017.05.016 28648983 PMC5539982

[15] Suh J , Kim NK , Shim W , Lee SH , Kim HJ , Moon E , et al. Mitochondrial fragmentation and donut formation enhance mitochondrial secretion to promote osteogenesis. Cell Metab. 2023;35:345–360.e7. 10.1016/j.cmet.2023.01.003 36754021

[16] Rodrigues T , Ferraz LS . Therapeutic potential of targeting mitochondrial dynamics in cancer. Biochem Pharmacol. 2020;182:114282. 10.1016/j.bcp.2020.114282 33058754

[17] Liu C , Han Y , Gu X , Li M , Du Y , Feng N , et al. Paeonol promotes Opa1‐mediated mitochondrial fusion via activating the CK2alpha‐Stat3 pathway in diabetic cardiomyopathy. Redox Biol. 2021;46:102098. 10.1016/j.redox.2021.102098 34418601 PMC8385203

[18] Cui LJ , Liu PS . Two types of contact between lipid droplets and mitochondria. Front Cell Dev Biol. 2020;8:618322. 10.3389/fcell.2020.618322 33385001 PMC7769837

[19] Kalia R , Wang RY , Yusuf A , Thomas PV , Agard DA , Shaw JM , et al. Structural basis of mitochondrial receptor binding and constriction by DRP1. Nature. 2018;558:401–405. 10.1038/s41586-018-0211-2 29899447 PMC6120343

[20] Wai T , Langer T . Mitochondrial dynamics and metabolic regulation. Trends Endocrinol Metab. 2016;27:105–117. 10.1016/j.tem.2015.12.001 26754340

[21] Kleele T , Rey T , Winter J , Zaganelli S , Mahecic D , Perreten Lambert H , et al. Distinct fission signatures predict mitochondrial degradation or biogenesis. Nature. 2021;593:435–439. 10.1038/s41586-021-03510-6 33953403

[22] Passmore JB , Carmichael RE , Schrader TA , Godinho LF , Ferdinandusse S , Lismont C , et al. Mitochondrial fission factor (MFF) is a critical regulator of peroxisome maturation. BBA‐Mol Cell Res. 2020;1867:118709. 10.1016/j.bbamcr.2020.118709 PMC7262603 32224193

[23] Jimah JR , Hinshaw JE . Structural insights into the mechanism of dynamin superfamily proteins. Trends Cell Biol. 2019;29:257–273. 10.1016/j.tcb.2018.11.003 30527453 PMC9623552

[24] Zhou BH , Wei SS , Jia LS , Zhang Y , Miao CY , Wang HW . Drp1/Mff signaling pathway is involved in fluoride‐induced abnormal fission of hepatocyte mitochondria in mice. Sci Total Environ. 2020;725:138192. 10.1016/j.scitotenv.2020.138192 32278173

[25] Lee JE , Westrate LM , Wu HX , Page C , Voeltz GK . Multiple dynamin family members collaborate to drive mitochondrial division. Nature. 2016;540:139–143. 10.1038/nature20555 27798601 PMC5656044

[26] Nagashima S , Tabara LC , Tilokani L , Paupe V , Anand H , Pogson JH , et al. Golgi‐derived PI(4)P‐containing vesicles drive late steps of mitochondrial division. Science. 2020;367:1366–1371. 10.1126/science.aax6089 32193326

[27] Pernas L , Scorrano L . Mito‐morphosis: mitochondrial fusion, fission, and cristae remodeling as key mediators of cellular function. Annu Rev Physiol. 2016;78:505–531. 10.1146/annurev-physiol-021115-105011 26667075

[28] Zhou Z , Torres M , Sha H , Halbrook CJ , Van den Bergh F , Reinert RB , et al. Endoplasmic reticulum‐associated degradation regulates mitochondrial dynamics in brown adipocytes. Science. 2020;368:54–60. 10.1126/science.aay2494 32193362 PMC7409365

[29] Castro‐Sepulveda M , Fernandez‐Verdejo R , Zbinden‐Foncea H , Rieusset J . Mitochondria‐SR interaction and mitochondrial fusion/fission in the regulation of skeletal muscle metabolism. Metabolism. 2023;144:155578. 10.1016/j.metabol.2023.155578 37164310

[30] Sabouny R , Shutt TE . Reciprocal regulation of mitochondrial fission and fusion. Trends Biochem Sci. 2020;45:564–577. 10.1016/j.tibs.2020.03.009 32291139

[31] Zacharioudakis E , Gavathiotis E . Mitochondrial dynamics proteins as emerging drug targets. Trends Pharmacol Sci. 2023;44:112–127. 10.1016/j.tips.2022.11.004 36496299 PMC9868082

[32] Archer SL . Mitochondrial dynamics – mitochondrial fission and fusion in human diseases. N Engl J Med. 2013;369:2236–2251. 10.1056/NEJMra1215233 24304053

[33] Jiang Y , Krantz S , Qin X , Li S , Gunasekara H , Kim YM , et al. Caveolin‐1 controls mitochondrial damage and ROS production by regulating fission – fusion dynamics and mitophagy. Redox Biol. 2022;52:102304. 10.1016/j.redox.2022.102304 35413643 PMC9018165

[34] Ashraf R , Kumar S . Mfn2‐mediated mitochondrial fusion promotes autophagy and suppresses ovarian cancer progression by reducing ROS through AMPK/mTOR/ERK signaling. Cell Mol Life Sci. 2022;79:573. 10.1007/s00018-022-04595-6 36308626 PMC11803038

[35] Wang Q , Yu P , Liu C , He X , Wang G . Mitochondrial fragmentation in liver cancer: emerging player and promising therapeutic opportunities. Cancer Lett. 2022;549:215912. 10.1016/j.canlet.2022.215912 36103914

[36] Xiong X , Hasani S , Young LEA , Rivas DR , Skaggs AT , Martinez R , et al. Activation of Drp1 promotes fatty acids‐induced metabolic reprograming to potentiate Wnt signaling in colon cancer. Cell Death Differ. 2022;29:1913–1927. 10.1038/s41418-022-00974-5 35332310 PMC9525627

[37] Padder RA , Bhat ZI , Ahmad Z , Singh N , Husain M . DRP1 promotes BRAF(V600E)‐driven tumor progression and metabolic reprogramming in colorectal cancer. Front Oncol. 2020;10:592130. 10.3389/fonc.2020.592130 33738242 PMC7961078

[38] Serasinghe MN , Wieder SY , Renault TT , Elkholi R , Asciolla JJ , Yao JL , et al. Mitochondrial division is requisite to RAS‐induced transformation and targeted by oncogenic MAPK pathway inhibitors. Mol Cell. 2015;57:521–536. 10.1016/j.molcel.2015.01.003 25658204 PMC4320323

[39] Thoudam T , Chanda D , Sinam IS , Kim BG , Kim MJ , Oh CJ , et al. Noncanonical PDK4 action alters mitochondrial dynamics to affect the cellular respiratory status. Proc Natl Acad Sci USA. 2022;119:e2120157119. 10.1073/pnas.2120157119 35969774 PMC9407676

[40] Huang CY , Chiang SF , Chen WT , Ke TW , Chen TW , You YS , et al. HMGB1 promotes ERK‐mediated mitochondrial Drp1 phosphorylation for chemoresistance through RAGE in colorectal cancer. Cell Death Dis. 2018;9:1004. 10.1038/s41419-018-1019-6 30258050 PMC6158296

[41] Inoue‐Yamauchi A , Oda H . Depletion of mitochondrial fission factor DRP1 causes increased apoptosis in human colon cancer cells. Biochem Biophys Res Commun. 2012;421:81–85. 10.1016/j.bbrc.2012.03.118 22487795

[42] Xie Y , Chen R , Yan L , Jia Z , Liang G , Wang Q . Transcription factor HOXC10 activates the expression of MTFR2 to regulate the proliferation, invasion and migration of colorectal cancer cells. Mol Med Rep. 2021;24:797. 10.3892/mmr.2021.12437 34523692 PMC8456344

[43] Shi L , Liu J , Peng Y , Zhang J , Dai X , Zhang S , et al. Deubiquitinase OTUD6A promotes proliferation of cancer cells via regulating Drp1 stability and mitochondrial fission. Mol Oncol. 2020;14:3169–3183. 10.1002/1878-0261.12825 33070427 PMC7718948

[44] Hu HF , Xu WW , Li YJ , He Y , Zhang WX , Liao L , et al. Anti‐allergic drug azelastine suppresses colon tumorigenesis by directly targeting ARF1 to inhibit IQGAP1‐ERK‐Drp1‐mediated mitochondrial fission. Theranostics. 2021;11:1828–1844. 10.7150/thno.48698 33408784 PMC7778598

[45] Locatelli L , Cazzaniga A , Fedele G , Zocchi M , Scrimieri R , Moscheni C , et al. A comparison of doxorubicin‐resistant colon cancer LoVo and leukemia HL60 cells: common features, different underlying mechanisms. Curr Issues Mol Biol. 2021;43:163–175. 10.3390/cimb43010014 34067290 PMC8929017

[46] Rainho MA , Siqueira PB , de Amorim ISS , Mencalha AL , Thole AA . Mitochondria in colorectal cancer stem cells – a target in drug resistance. Cancer Drug Resist. 2023;6:273–283. 10.20517/cdr.2022.116 37457136 PMC10344721

[47] Sun K , Chen L , Li Y , Huang B , Yan Q , Wu C , et al. METTL14‐dependent maturation of pri‐miR‐17 regulates mitochondrial homeostasis and induces chemoresistance in colorectal cancer. Cell Death Dis. 2023;14:148. 10.1038/s41419-023-05670-x 36810285 PMC9944299

[48] Barisciano G , Leo M , Muccillo L , Pranzini E , Parri M , Colantuoni V , et al. The miR‐27a/FOXJ3 Axis dysregulates mitochondrial homeostasis in colorectal cancer cells. Cancers (Basel). 2021;13:4994. 10.3390/cancers13194994 34638478 PMC8507763

[49] Rosolen D , Nunes‐Souza E , Marchi R , Tofolo MV , Antunes VC , Berti FCB , et al. MiRNAs action and impact on mitochondria function, metabolic reprogramming and chemoresistance of cancer cells: a systematic review. Biomedicine. 2023;11:693. 10.3390/biomedicines11030693 PMC10045760 36979672

[50] Dai W , Wang G , Chwa J , Oh ME , Abeywardana T , Yang Y , et al. Mitochondrial division inhibitor (mdivi‐1) decreases oxidative metabolism in cancer. Br J Cancer. 2020;122:1288–1297. 10.1038/s41416-020-0778-x 32147668 PMC7188673

[51] Yakobov S , Dhingra R , Margulets V , Dhingra A , Crandall M , Kirshenbaum LA . Ellagic acid inhibits mitochondrial fission protein Drp‐1 and cell proliferation in cancer. Mol Cell Biochem. 2023;478:2029–2040. 10.1007/s11010-022-04627-6 36607523

[52] Chen M , Ye K , Zhang B , Xin Q , Li P , Kong AN , et al. Paris saponin II inhibits colorectal carcinogenesis by regulating mitochondrial fission and NF‐kappaB pathway. Pharmacol Res. 2019;139:273–285. 10.1016/j.phrs.2018.11.029 30471409 PMC8108001

[53] Tailor D , Hahm ER , Kale RK , Singh SV , Singh RP . Sodium butyrate induces DRP1‐mediated mitochondrial fusion and apoptosis in human colorectal cancer cells. Mitochondrion. 2014;16:55–64. 10.1016/j.mito.2013.10.004 24177748 PMC4004730

[54] Zhang BY , Zhang L , Chen YM , Qiao X , Zhao SL , Li P , et al. Corosolic acid inhibits colorectal cancer cells growth as a novel HER2/HER3 heterodimerization inhibitor. Br J Pharmacol. 2021;178:1475–1491. 10.1111/bph.15372 33443775

[55] Zinecker H , Ouaret D , Ebner D , Gaidt MM , Taylor S , Aulicino A . ICG‐001 affects DRP1 activity and ER stress correlative with its anti‐proliferative effect. Oncotarget. 2017;8:106764–106777. 10.18632/oncotarget.22264 29290987 PMC5739772

[56] Qin Y , Yu Y , Yang C , Wang Z , Yang Y , Wang C , et al. Atractylenolide I inhibits NLRP3 Inflammasome activation in colitis‐associated colorectal cancer via suppressing Drp1‐mediated mitochondrial fission. Front Pharmacol. 2021;12:674340. 10.3389/fphar.2021.674340 34335248 PMC8320763

[57] Zamorano‐Leon JJ , Ballesteros S , de Las Heras N , Alvarez‐Sala L , de la Serna‐Soto M , Zekri‐Nechar K , et al. Effect of pectin on the expression of proteins associated with mitochondrial biogenesis and cell senescence in HT29‐human colorectal adenocarcinoma cells. Prev Nutr Food Sci. 2019;24:187–196. 10.3746/pnf.2019.24.2.187 31328124 PMC6615348

[58] Wang SQ , Cui SX , Qu XJ . Metformin inhibited colitis and colitis‐associated cancer (CAC) through protecting mitochondrial structures of colorectal epithelial cells in mice. Cancer Biol Ther. 2019;20:338–348. 10.1080/15384047.2018.1529108 30359174 PMC6370369

[59] Yang M , Liu Q , Dai M , Peng R , Li X , Zuo W , et al. FOXQ1‐mediated SIRT1 upregulation enhances stemness and radio‐resistance of colorectal cancer cells and restores intestinal microbiota function by promoting beta‐catenin nuclear translocation. J Exp Clin Cancer Res. 2022;41:70. 10.1186/s13046-021-02239-4 35183223 PMC8857837

[60] Liao M , Sun X , Zheng W , Wu M , Wang Y , Yao J , et al. LINC00922 decoys SIRT3 to facilitate the metastasis of colorectal cancer through up‐regulation the H3K27 crotonylation of ETS1 promoter. Mol Cancer. 2023;22:163. 10.1186/s12943-023-01859-y 37789393 PMC10548613

[61] Wang Y , Sun X , Ji K , Du L , Xu C , He N , et al. Sirt3‐mediated mitochondrial fission regulates the colorectal cancer stress response by modulating the Akt/PTEN signalling pathway. Biomed Pharmacother. 2018;105:1172–1182. 10.1016/j.biopha.2018.06.071 30021354

[62] Bao D , Zhao J , Zhou X , Yang Q , Chen Y , Zhu J , et al. Mitochondrial fission‐induced mtDNA stress promotes tumor‐associated macrophage infiltration and HCC progression. Oncogene. 2019;38:5007–5020. 10.1038/s41388-019-0772-z 30894684 PMC6755992

[63] Chen Y , Yang J , Zuo Y , Zhang C , Pu Y , Ren Q , et al. Voacamine is a novel inhibitor of EGFR exerting oncogenic activity against colorectal cancer through the mitochondrial pathway. Pharmacol Res. 2022;184:106415. 10.1016/j.phrs.2022.106415 36029932

[64] Sun Y , Yang YM , Hu YY , Ouyang L , Sun ZH , Yin XF , et al. Inhibition of nuclear deacetylase Sirtuin‐1 induces mitochondrial acetylation and calcium overload leading to cell death. Redox Biol. 2022;53:102334. 10.1016/j.redox.2022.102334 35636016 PMC9142701

[65] Zhuo F‐F , Li L , Liu T‐T , Liang X‐M , Yang Z , Zheng Y‐Z , et al. Lycorine promotes IDH1 acetylation to induce mitochondrial dynamics imbalance in colorectal cancer cells. Cancer Lett. 2023;573:216364. 10.1016/j.canlet.2023.216364 37648148

[66] Li H , He F , Zhao X , Zhang Y , Chu X , Hua C , et al. YAP inhibits the apoptosis and migration of human rectal cancer cells via suppression of JNK‐Drp1‐mitochondrial fission‐HtrA2/Omi pathways. Cell Physiol Biochem. 2017;44:2073–2089. 10.1159/000485946 29241219

[67] Yao W , Zhu S , Li P , Zhang S . Large tumor suppressor kinase 2 overexpression attenuates 5‐FU‐resistance in colorectal cancer via activating the JNK‐MIEF1‐mitochondrial division pathway. Cancer Cell Int. 2019;19:97. 10.1186/s12935-019-0812-3 31011291 PMC6460675

[68] Zhang Y , Wang M , Xu X , Liu Y , Xiao C . Matrine promotes apoptosis in SW480 colorectal cancer cells via elevating MIEF1‐related mitochondrial division in a manner dependent on LATS2‐hippo pathway. J Cell Physiol. 2019;234:22731–22741. 10.1002/jcp.28838 31119752

[69] Kim YY , Yun SH , Yun J . Downregulation of Drp1, a fission regulator, is associated with human lung and colon cancers. Acta Biochim Biophys Sin (Shanghai). 2018;50:209–215. 10.1093/abbs/gmx137 29329364

[70] Qian J , Fang D , Lu H , Cao Y , Zhang J , Ding R , et al. Tanshinone IIA promotes IL2‐mediated SW480 colorectal cancer cell apoptosis by triggering INF2‐related mitochondrial fission and activating the Mst1‐Hippo pathway. Biomed Pharmacother. 2018;108:1658–1669. 10.1016/j.biopha.2018.09.170 30372868

[71] Jieensinue S , Zhu H , Li G , Dong K , Liang M , Li Y . Tanshinone IIA reduces SW837 colorectal cancer cell viability via the promotion of mitochondrial fission by activating JNK‐Mff signaling pathways. BMC Cell Biol. 2018;19:21. 10.1186/s12860-018-0174-z 30253740 PMC6157045

[72] Zhang K , Zhang D , Wang J , Wang Y , Hu J , Zhou Y , et al. Aloe gel glucomannan induced colon cancer cell death via mitochondrial damage‐driven PINK1/Parkin mitophagy pathway. Carbohydr Polym. 2022;295:119841. 10.1016/j.carbpol.2022.119841 35989033

[73] Liskova V , Kajsik M , Chovancova B , Roller L , Krizanova O . Camptothecin, triptolide, and apoptosis inducer kit have differential effects on mitochondria in colorectal carcinoma cells. FEBS Open Bio. 2022;12:913–924. 10.1002/2211-5463.13401 PMC9063445 35318813

[74] He Y , Kan W , Li Y , Hao Y , Huang A , Gu H , et al. A potent and selective small molecule inhibitor of myoferlin attenuates colorectal cancer progression. Clin Transl Med. 2021;11:e289. 10.1002/ctm2.289 33634965 PMC7868085

[75] Zhang B , Liu Q , Wen W , Gao H , Wei W , Tang A , et al. The chromatin remodeler CHD6 promotes colorectal cancer development by regulating TMEM65‐mediated mitochondrial dynamics via EGF and Wnt signaling. Cell Discov. 2022;8:130. 10.1038/s41421-022-00478-z 36473865 PMC9727023

[76] Cai WF , Zhang C , Wu YQ , Zhuang G , Ye Z , Zhang CS , et al. Glutaminase GLS1 senses glutamine availability in a non‐enzymatic manner triggering mitochondrial fusion. Cell Res. 2018;28:865–867. 10.1038/s41422-018-0057-z 29934617 PMC6082853

[77] Li L , Chen Q , Yu Y , Chen H , Lu M , Huang Y , et al. RKI‐1447 suppresses colorectal carcinoma cell growth via disrupting cellular bioenergetics and mitochondrial dynamics. J Cell Physiol. 2020;235:254–266. 10.1002/jcp.28965 31237697

[78] Lin LT , Shi YC , Choong CY , Tai CJ . The fruits of *Paris polyphylla* inhibit colorectal cancer cell migration induced by *Fusobacterium nucleatum*‐derived extracellular vesicles. Molecules. 2021;26:4081. 10.3390/molecules26134081 34279421 PMC8271733

[79] Liu W , Chen S , Xie W , Wang Q , Luo Q , Huang M , et al. MCCC2 is a novel mediator between mitochondria and telomere and functions as an oncogene in colorectal cancer. Cell Mol Biol Lett. 2023;28:80. 10.1186/s11658-023-00487-0 37828426 PMC10571261

[80] Wang SQ , Yang XY , Cui SX , Gao ZH , Qu XJ . Heterozygous knockout insulin‐like growth factor‐1 receptor (IGF‐1R) regulates mitochondrial functions and prevents colitis and colorectal cancer. Free Radic Biol Med. 2019;134:87–98. 10.1016/j.freeradbiomed.2018.12.035 30611867

[81] Leo M , Muccillo L , Pranzini E , Barisciano G , Parri M , Lopatriello G , et al. Transcriptomic analysis of colorectal cancer cells treated with oil production waste products (OPWPs) reveals enrichment of pathways of mitochondrial functionality. Cell. 2022;11:3992. 10.3390/cells11243992 PMC9776412 36552757

[82] Kuznetsov AV , Javadov S , Margreiter R , Grimm M , Hagenbuchner J , Ausserlechner MJ . Structural and functional remodeling of mitochondria as an adaptive response to energy deprivation. Biochim Biophys Acta Bioenerg. 2021;1862:148393. 10.1016/j.bbabio.2021.148393 33549532 PMC9022200

[83] Li J , Ye Y , Liu Z , Zhang G , Dai H , Li J , et al. Macrophage mitochondrial fission improves cancer cell phagocytosis induced by therapeutic antibodies and is impaired by glutamine competition. Nat Cancer. 2022;3:453–470. 10.1038/s43018-022-00354-5 35484420

[84] Wang Y , Subramanian M , Yurdagul A Jr , Barbosa‐Lorenzi VC , Cai B , de Juan‐Sanz J , et al. Mitochondrial fission promotes the continued clearance of apoptotic cells by macrophages. Cell. 2017;171:331–345.e22. 10.1016/j.cell.2017.08.041 28942921 PMC5679712

[85] Mitochondrial fission in macrophages fuels phagocytosis of tumor cells. Nat Cancer. 2022;3:384–385. 10.1038/s43018-022-00350-9 35484421

[86] Ding C , Shrestha R , Zhu X , Geller AE , Wu S , Woeste MR , et al. Inducing trained immunity in pro‐metastatic macrophages to control tumor metastasis. Nat Immunol. 2023;24:239–254. 10.1038/s41590-022-01388-8 36604547 PMC10636755

[87] Buck MD , O'Sullivan D , Klein Geltink RI , Curtis JD , Chang CH , Sanin DE , et al. Mitochondrial dynamics controls T cell fate through metabolic programming. Cell. 2016;166:63–76. 10.1016/j.cell.2016.05.035 27293185 PMC4974356

[88] He J , Shangguan X , Zhou W , Cao Y , Zheng Q , Tu J , et al. Glucose limitation activates AMPK coupled SENP1‐Sirt3 signalling in mitochondria for T cell memory development. Nat Commun. 2021;12:4371. 10.1038/s41467-021-24619-2 34272364 PMC8285428

[89] Herkenne S , Ek O , Zamberlan M , Pellattiero A , Chergova M , Chivite I , et al. Developmental and tumor angiogenesis requires the mitochondria‐shaping protein Opa1. Cell Metab. 2020;31:987–1003.e8. 10.1016/j.cmet.2020.04.007 32315597

